# Effects of Creep Feed Provision on Behavior and Performance of Piglets Around Weaning

**DOI:** 10.3389/fvets.2020.520035

**Published:** 2020-11-12

**Authors:** Anouschka Middelkoop, Raka Choudhury, Walter J. J. Gerrits, Bas Kemp, Michiel Kleerebezem, J. Elizabeth Bolhuis

**Affiliations:** ^1^Adaptation Physiology Group, Department of Animal Sciences, Wageningen University & Research, Wageningen, Netherlands; ^2^Host-Microbe Interactomics Group, Department of Animal Sciences, Wageningen University & Research, Wageningen, Netherlands; ^3^Animal Nutrition Group, Department of Animal Sciences, Wageningen University & Research, Wageningen, Netherlands

**Keywords:** feeding strategies, feed intake, fiber, growth, nutrition, pig, stress, suckling

## Abstract

Creep feed provision may ease weaning, hence we determined the impact of providing fibrous creep feed before weaning and adding this feed to the post-weaning diet on piglet behavior and performance. Pre-weaning, litters with on average 12 piglets were given creep feed (CF, *n* = 12 litters) or not (NF, *n* = 10 litters). Post-weaning, piglets (*n* = 8 pens with 4 piglets/treatment) were given a weaner diet (CON) or weaner diet supplemented with creep feed (CS). Behaviors were scored in the home pen at d11, 16, 22 and 27 after birth and at wk 1 and 2 post-weaning. Feed intake, growth and fecal consistency were measured up to d14 post-weaning. A blood sample was taken at d2, 15 and 29 after birth and d2, 5 and 14 post-weaning. CF-piglets consumed on average 397 ± 71 g creep feed before weaning. CF-piglets grew faster in the last week before weaning than NF-piglets (249 ± 7 vs. 236 ± 11 g/d, *F*_(1, 18)_ = 5.81, *P* = 0.03). However, CF- and NF-piglets did not differ in weaning weight, within-litter coefficient of variation in weaning weight, behaviors in the farrowing and weaner pen, and haptoglobin concentrations. Creep feed supplementation enhanced feed exploration at wk 2 post-weaning (0.29 ± 0.08 vs. 0.11 ± 0.03%, *F*_(1, 27)_ = 5.27, *P* = 0.03), but did not affect other post-weaning behaviors. Pre-weaning creep feed provision and post-weaning creep feed supplementation did not affect overall feed intake, growth, feed efficiency and fecal consistency for the first 14 days post-weaning, neither body weight at d14 post-weaning. Nevertheless, CF-piglets had a lower within-pen coefficient of variation in body weight at d14 post-weaning than NF-piglets (13.6 ± 1.9 vs. 15.1 ± 1.5%, *F*_(1, 26)_ = 6.89, *P* = 0.01). In conclusion, pre-weaning creep feed provision and post-weaning creep feed supplementation had no clear effects on piglet behavior and performance.

## Introduction

Weaning in piglets often results in stress [reviewed by (1)], temporary fasting and in a low feed intake [e.g. (2, 3)]. Consequently, piglets are predisposed to maldigestion and malabsorption, colonization by intestinal pathogens, growth stasis, diarrhea [reviewed by (4, 5)] and behaviors associated with weaning stress such as aggression, manipulation of ears and tails, belly nosing, and vocalizing (6, 7). Also acute phase proteins, including haptoglobin, are elevated after weaning compared to pre-weaning values (8, 9) and values of unweaned piglets (9) and are therefore considered biomarkers for stress, acute infection, and inflammation. An irregular feed intake pattern with periods of fasting and a low post-weaning feed intake increases haptoglobin in pigs (10, 11) and haptoglobin is inversely related to individual body weight gain in the post-weaning period (10, 12). This indicates the importance of a short latency to eat and regular feed intake pattern after weaning, as piglets will feel less hungry and have less intestinal dysbiosis, which can both affect stress levels and behaviors (13).

Weaning in commercial conditions includes multiple simultaneous stressors, including maternal separation and introduction to non-littermate piglets (social stress), relocation to unfamiliar housing (environmental stress), and a change in diet from sow's milk to solid feed (nutritional stress). Reducing the nutritional stressor at weaning may help to migitate weaning-related problems by habituating piglets to solid feed as an alternative energy source while they are with the sow (14), which may reduce neophobia for the weaner diet (13, 15). Consequently, this may improve the feed intake and hence growth of newly weaned piglets, although the impact on these performance parameters seems inconsistent [positive effects: (14, 16), negative effects: (17), lack of effect: (18, 19)]. A higher post-weaning feed intake may reduce stress levels (13), but a reduction in stress biomarkers at weaning as a result of creep feed provision is not yet confirmed (20). If creep feed provision reduces weaning stress, it may also affect post-weaning piglet behavior, but to our knowledge this has not been studied. Apart from a potential effect on post-weaning behavior, creep feed intake could also affect pre-weaning behavior as it may derive from exploration (21–23). Moreover, supplementing the creep feed with dietary fibers may affect pre-weaning piglet behavior, including activity, exploration, and interaction with littermates (24, 25). Only a single study investigated the impact of creep feed provision on pre-weaning behavior, focusing on suckling, sleeping and fighting, and found no effects (26). In addition, supplementing the creep feed with dietary fibers increased large intestinal size, fill and volatile fatty acid production before being weaned (27), which may elicit health and growth benefits when piglets are subjected to the weaner diet. Early familiarization with (fibrous) feed may thus help piglets to cope with weaning. This seems particularly the case when the same diet is given before and after weaning (28), however it remains unknown whether a small quantity of the pre-weaning diet on top of the weaner diet can also reduce post-weaning food neophobia.

Therefore, the present study aimed to determine the impact of fibrous creep feed provision, the impact of adding this creep feed in a small quantity to the weaner diet, and the interaction between pre-weaning creep feed provision and post-weaning creep feed supplementation on piglet behavior and performance after weaning. We hypothesized that creep feed provision would stimulate feed intake and growth of weaned piglets, and reduce haptoglobin concentrations and behaviors associated with weaning stress. We expected additional beneficial effects on the performance of weaned piglets that had access to creep feed before and, as supplement on top of the weaner diet, after weaning, as these piglets will likely experience the lowest level of food neophobia.

## Methods

The Animal Care and Use committee of Wageningen University & Research (Wageningen, The Netherlands) approved the protocol of the experiment (AVD104002016515). The protocol is in accordance with the Dutch law on animal experimentation, which complies with the European Directive 2010/63/EU on the protection of animals used for scientific purposes.

### Animals, Housing, and Management

**Figure 1:**
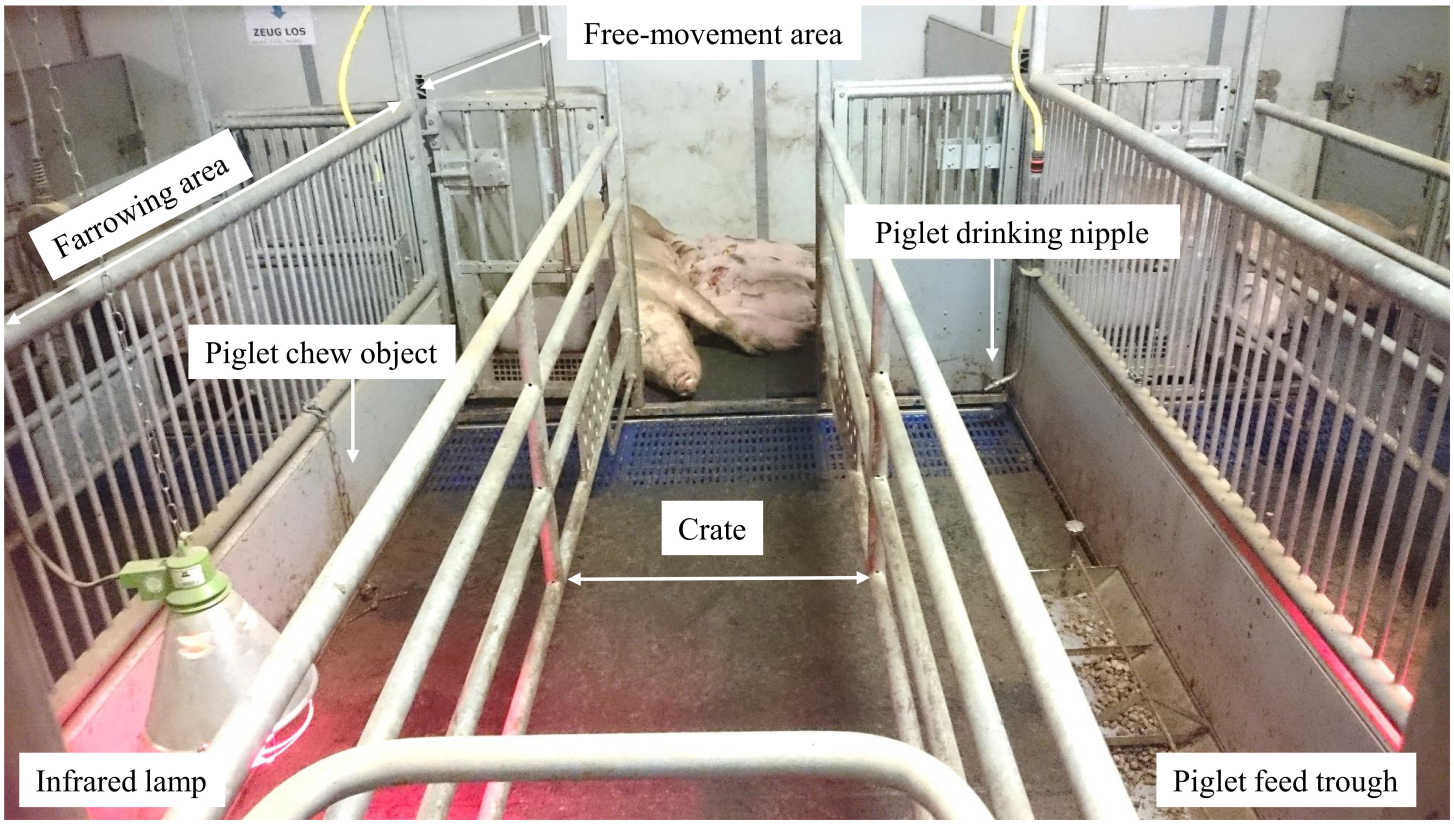
Set-up of the farrowing pen, consisting of a farrowing and free-movement area. Piglets had access to a piglet feed trough from 2 days of age, either with or without creep feed (see treatments).

Twenty-two multiparous Topigs-20 sows (range parity: 3–5) were housed and inseminated at research facility Carus (Wageningen University & Research, The Netherlands) in two consecutive batches, with *n* = 10 and *n* = 12 sows, respectively. Sows were fed commercially available diets (“Inno Dracht” during gestation and “Inno Lac Vital” during lactation, Coppens Diervoeding, Helmond, The Netherlands) twice a day, at 7:30 and 16.00 h. One week before farrowing, the sows were moved to two adjacent farrowing rooms and were individually housed. The pen (Figure 1) was equipped with a farrowing area (2.85 × 1.80 m, 80% mats and 20% slatted area) and a free-movement area (1.85 × 1.80 m, 100% mats). The farrowing area consisted of a crate (2.85 × 0.60 m) that included a feed trough, drinking nipple and metal chain with bolts as chew object for the sow. The sow was fixed in the crate when showing impending signs of farrowing until four days post-partum to minimize piglet crushing. After that, the sow could move from her crate to the free-movement area and back. The free-movement area included a drinking nipple for the sow. Around farrowing sows were provided a jute sack as nesting material. Sows were fed in the feed trough of the crate, and from d2 after farrowing sows were fed in a removable feed trough in the free-movement area, while being separated for 30 min from their piglets which stayed in the farrowing area. Unconsumed sow feed was removed after feeding, before piglets were given access to the free-movement area again, to prevent piglets from eating sow feed. Within 24 h after birth, piglets were weighed, ear tagged and injected intramuscularly with iron. Litter size varied from 9 to 14 piglets per litter. The farrowing area had an infrared lamp, drinking nipple and chew object for the piglets. The chew object was a metal chain with bolts given from 1 week of age. From 2 days of age (fixed to birth date) a stainless steel feed trough (100 × 24 × 8 cm, MS Schippers, Bladel, The Netherlands), which had approximately eight feeding places for the piglets, was placed in the farrowing area. Room temperature was 25°C around farrowing and was gradually decreased to 22°C until weaning.

A subset of 128 piglets was weaned on the same day at 4 weeks of age (29.8 ± 0.1 days of age) in two equal batches and relocated in two adjacent weaner rooms per batch in which they were housed until 6 weeks of age. At weaning, piglets were mixed with piglets within pre-weaning treatment and housed in groups of four unfamiliar piglets, of which two males and two females, in a pen of 1.80 × 2.85 m with 80% mats and 20% slatted floor. The weaner pen was equipped with a feeder (12 × 50 cm) having three feeding spaces, a drinking trough, infrared lamp and chew object, which was a metal chain with bolts. Additional chew objects were provided and replaced daily in a cycle of four objects: a squeaky ball or metal chain with two solid balls, PVC pipe or hose attached. Piglets had *ad libitum* access to a commercial weaner diet (2-mm pellet, 16.4% crude protein, 4.4% crude fiber and 11.9 g standardized ileal digestible lysine/kg dry matter, “Inno Speen Pro,” Coppens Diervoeding, Helmond, The Netherlands). Room temperature was kept at 25°C in the first week after weaning and decreased to 23°C in the second week. In both farrowing and weaner rooms, lights and radio were on between 07:00 and 19:00 h and lighting was dimmed during the night to allow video recordings for farrowing time and other research purposes.

### Treatments

Piglets were assigned to one of four treatment combinations in a 2 × 2 arrangement, with pre-weaning creep feed provision and post-weaning creep feed supplementation as experimental factors.

#### Pre-weaning Creep Feed Provision

From 2 days of age twelve litters (*n* = 6/batch) were provided with creep feed in the piglet feed trough (**CF**) and 10 litters (*n* = 4 and *n* = 6 in batch 1 and 2, respectively) were not provided with creep feed before weaning and the trough was kept empty (**NF**). The creep feed (11.8 MJ/kg as-fed net energy, 195 g crude protein, 11.9 g standardized ileal digestible lysine/kg dry matter) was high in dietary non-starch polysaccharides (261 g/kg dry matter), originating from cereal grains, sugarbeet pulp, oat hulls, galactooligosaccharide, inulin and high-amylose starch (Tables 1, 2). The amount of creep feed in the feed trough was checked at least twice daily to provide the creep feed *ad libitum* and all creep feed was replaced at each feed weigh-back to maintain freshness of the feed. The creep feed was mixed by Research Diet Services (Wijk bij Duurstede, The Netherlands) and extruded using a co-rotating double screw extruder (M.P.F. 50, Baker Perkins, Peterborough, United Kingdom). Extruder settings intendedly varied during production, resulting in differences in pellet diameter (2, 8 and 14 mm), length (10 mm for 2-mm diameter pellet, 8 and 22 mm for 8-mm diameter pellet and 10 and 20 mm for 14-mm diameter pellet, respectively), texture and hardness (7.3–17.7 kg) to create dietary diversity. The different pellet types were mixed evenly and provided as one diet. To stimulate early uptake of creep feed (30, 31), sows were allowed to eat creep feed together with their piglets during the first week of creep feed provision two times a day for 10–20 min. Immediately after sow feeding, 250 g of creep feed/pen was provided in the piglet feed through to which sows were given temporary access. The consumption of creep feed by the sows was not included in the assessment of piglets' creep feed intake. To compensate for the creep feed eaten by sows with CF-litters, sows with NF-litters received 250 g of creep feed per feeding (i.e., 500 g per day) additional to their lactation diet. Due to variation in creep feed intake by sows, even after habituation to the creep feed, we decided to stop facilitation of eating behavior by the sow. Therefore, all sows in batch 2 received the creep feed supplement on top of their lactation feed.

**Table 1:** Nutrient profile of the creep feed.

**Calculated nutrient composition^a^**	**Creep feed**
Net energy	11.8
Dry matter	891
Starch	290
Non-starch polysaccharides^b^	261
Crude protein	195
Crude fat	61
Crude fiber	44
Crude ash	57
Calcium	9.1
Phosphorus	6.1
Sodium	2.2
Standardized ileal digestible lysine	11.9
Standardized ileal digestible methionine	4.8
Standardized ileal digestible threonine	7.1
Standardized ileal digestible tryptophan	2.4

a *According to CVB (29). Nutrients are presented in g/kg dry matter, except for dry matter (g/kg) and net energy (MJ/kg)*.

b *Calculated as the difference between dry matter and the sum of starch, sugars, crude protein, crude fat and crude ash*.

**Table 2:** Ingredient composition of the creep feed.

**Ingredient component**	**%**
Wheat	21.9
Barley	15
Maize	15
Soy protein concentrate	7
Soybeans (heat treated)	5
Galacto-oligosaccharides	5
Potato protein	4
Sugarbeet pulp (dehydrated)	4
Oat hulls	4
Inulin	4
High-amylose starch (± 75% amylose)	4
Soybean oil	3
Blood meal (spray dried)	2
Dicalcium phosphate	1.7
Sucrose	1.5
Calcium carbonate	1.0
Sodium chloride	0.5
Premix^a^	0.5
Potassium bicarbonate	0.3
L-lysine hydrochloride	0.3
DL-methionine	0.2
L-threonine	0.04
L-tryptophan	0.04
Total	100

a *Vitamin and mineral premix (per kg of feed): vitamin A: 10000 IU, vitamin D3: 2000 IU, vitamin E: 40 mg, vitamin K: 1.5 mg, vitamin B1: 1 mg, vitamin B2: 4 mg, vitamin B6: 1.5 mg, vitamin B12: 0.02 mg, niacin: 30 mg, D-pantothenic acid: 15 mg, choline chloride: 150 mg, folate: 0.4 mg, biotin: 0.05 mg, iron: 100 mg, copper: 20 mg, manganese: 30 mg, zinc: 70 mg, iodine: 0.7 mg, selenium: 0.25 mg, anti-oxidant: 125 mg*.

Distribution of sows over the farrowing rooms was balanced for sow body weight and back fat at 1 week before farrowing. At 1 week before farrowing, sows were initially allocated to the treatment groups in such a way that the average parity was the same for both treatments. There were a few extra sows (reserves) and at the start of the experiment at d2 some of the sows and their litters were excluded due to antibiotic treatment of the sow or a low litter size (<9 piglets alive per litter at d2). As it could not be anticipated beforehand which sows would be excluded from the experiment, distribution of treatment groups over the farrowing rooms was not perfectly balanced in batch 2 (4:2 distribution per room). Within the farrowing rooms, there were 2 blocks of 4 adjacent pens, with each block consisting of the two treatment groups. The first and last pen of the rooms were kept empty whenever possible (Supplementary Table S1). Allocation of the remaining litters to one of the two treatment groups was done at d2 and was based on 1) average body weight (BW) of the litter at d0 (CF: 1.37 ± 0.04 vs. NF: 1.38 ± 0.05) and d2 (CF: 1.53 ± 0.06 vs. NF: 1.52 ± 0.05), 2) birth date, and 3) sow's parity (range: 3–5, CF: 4.3 ± 0.2 vs. NF: 4.3 ± 0.2). Weaning age (maximum range of 5 days between litters) did not differ between the treatment groups (CF: 29.9 ± 0.43 vs. NF: 29.7 ± 0.52 days of age). CF-litters had 12.8 ± 0.4 piglets and NF-litters had 11.7 ± 0.6 piglets at the start of the treatments at 2 days of age, while CF-litters had 11.8 ± 0.5 and NF-litters had 11.4 ± 0.5 piglets/litter at weaning.

#### Post-weaning Creep Feed Supplementation

After weaning, effects of creep feed provision before weaning and creep feed supplementation after weaning were studied in a 2 × 2 arrangement. Thus, 16 pens of which 8 with CF- and 8 with NF-piglets had *ad libitum* access to the weaner diet (**CON**), and 16 pens of which 8 with CF- and 8 with NF-piglets received, on top of their weaner diet that was provided *ad libitum*, a limited amount of 80 g of creep feed per pen twice a day as supplementation (**CS**). The creep feed supplement was provided in the same feeder as the weaner diet.

Piglets were selected based on their sex and their BW at one day before weaning (CF-CON: 8.35 ± 0.09 and CF-CS: 8.34 ± 0.09 vs. NF-CON: 7.91 ± 0.11 kg and NF-CS: 7.91 ± 0.07 for selected piglets), which was close to the average weight of the litter and treatment group (CF: 8.27 ± 0.20 vs. NF: 7.85 ± 0.31 for all suckling piglets). The selected piglets originated from ten litters per treatment group (from 10 out of 12 CF-litters and 10 out of 10 NF-litters to keep genetic variation the same between treatments). As the number of NF-litters was lower in batch 1 (*n* = 4) than in batch 2 (*n* = 6), this implies that 8 piglets/litter were selected in batch 1 and 4–6 piglets/litter were selected in batch 2 for both treatments. Piglets with a history of medication and leg/claw problems were excluded from selection. Distribution of treatment groups over the weaner rooms was balanced, with 2 pens/treatment per weaner room. Within rooms, there were 2 blocks of 4 adjacent pens, with each block consisting of the 4 treatment groups (Supplementary Table S2).

### Measurements

A timeline of all measurements is provided in Supplementary Figure S1.

#### Piglet Performance

Piglets were individually weighed on d2 (fixed to birth date, thereafter all measurements were performed on the same day for all piglets), 15, 21, 29 after birth and d2, 5, 9 and 14 post-weaning. Creep feed intake was determined per CF-litter between d2-15, 15-21, and 21-30 after birth. Post-weaning feed intake was determined per pen between d0-2, 2-5, 5-9, and 9-14 after weaning. The intake per feed type (weaner feed or creep feed supplement) was also determined. If any, feed remains on the floor were collected. Feed wastage was kept to a minimum by placing the feeders on the solid floor in the farrowing and weaner pens. Fecal consistency scores of fecal droppings in the pens were taken daily by one observer for the first 14 days post-weaning according to the fecal classification scale with four categories of Pedersen and Toft (32). According to this scale score 1 (firm and shaped) and 2 (soft and shaped) represent normal feces, and were therefore combined into one score before data analysis. Score 3 (loose) and 4 (watery) represent diarrhea. The highest fecal consistency score that was observed in a pen was selected on each measurement day and averaged over 2 weeks post-weaning to calculate the mean fecal consistency score (FCS) per pen.

#### Piglet Behavior

Piglets were individually numbered using dark permanent hair dye (pre-weaning) or animal marking spray (post-weaning) to allow individual recognition during behavioral observations. Live behavioral observations were done on all piglets in the farrowing rooms at d11, 16, 22 and 27 after birth using 4.5-min instantaneous scan sampling for six sessions of 63 min/d, i.e., 84 scans/piglet/d. Observation sessions started at 8:15, 9:30, 10:45, 13:45, 15:00, and 16:45 h. Behavior in the weaner rooms was observed at d6 and 13 post-weaning using 2-min instantaneous scan sampling for six sessions of 1h/d, i.e., 180 scans/piglet/d, starting at 8:00, 9:15, 10:30, 14:00, 15:15, and 16:30 h. Behaviors were scored using a Psion hand-held computer with the Pocket Observer 3.1 software package (Noldus Information Technology, Wageningen, The Netherlands) or using a pen and scoring sheets. The ethograms are given in Supplementary Table S3. Before weaning six, and after weaning two, well-trained observers scored the behaviors from the corridor adjacent to the pens. Observers were always balanced over treatments and changed rooms every hour.

#### Haptoglobin Concentrations

Haptoglobin was determined at d29 after birth in 21 CF- and 20 NF-piglets. In 28 of these piglets additional samples were taken before weaning at d2 and 15 after birth (1-2 pigs per litter, *n* = 14 per treatment) and/or after weaning in 14 CF-CON and 14 NF-CON-piglets at d2, 5 and 14 post-weaning (1-2 pigs per pen). Piglets originated from 10 litters/treatment. Male piglets without a history of medical treatment or leg/claw problems were selected. Piglets were selected from one sex only to minimize within-treatment variation, although it should be noted that the concentration of haptoglobin does not seem to differ between male and female piglets at this age (33). Blood was drawn by puncture of the jugular vein and the order of sampling was balanced for treatment and room. Blood was collected in VACUETTE® K3EDTA tubes (Greiner Bio-One, Alphen aan den Rijn, The Netherlands), subsequently kept on a layer of ice, and centrifuged at 1,300 g for 10 min at 4°C to separate plasma. Plasma was stored at −20°C until analysis of the haptoglobin concentration using a commercial kit (PHASE™ Haptoglobin Assay, Tridelta Development Limited, Maynooth, Ireland). According to the kit test procedure, hemoglobin reagent (100 μL) was added to sera (7.5 μL), gently mixed, followed by addition of chromogen reagent (140 μL). The solution was incubated for 5 min at room temperature and the absorbance was read immediately at 600 nm in a microplate reader. The concentration of haptoglobin (mg/mL plasma) was calculated with a standard linear curve for known concentrations of haptoglobin.

### Statistical Analyses

#### Data Processing

Piglet behaviors in the farrowing room at d11, 16, 22 and 27 after birth were averaged per piglet per day (84 scans) and expressed as proportions of time. Based on the observations in the farrowing rooms we also discriminated “eaters,” i.e., piglets scored eating creep feed from the feed trough or floor at least once, from “non-eaters” per observation day. Piglet behaviors in the weaner room at d6 and 13 after weaning were averaged per piglet per day (180 scans) and expressed as proportions of time.

Weighing piglets at d2 and the provision of creep feed at d2 were fixed to the birth date of the litter, thereafter all measurements were performed on the same day for all piglets. Growth and creep feed intake between d2-15 before weaning consisted of a different number of days per litter as result of the variation in birth date. This was accounted for by calculating average daily gain and average daily creep feed intake. Three of the selected male weaner piglets, i.e., 1 CF-CON and 2 NF-CON piglets, were excluded from haptoglobin analyses due to health issues, as haptoglobin increases with acute infection and inflammation.

#### Data Analysis

Data were analyzed using the statistical software SAS 9.4 (SAS Institute Inc., Cary, NC, USA). An overview of data analysis, i.e., the statistical models used for the different response parameters, can be found in Supplementary Table S4. Model residuals of the general linear (mixed) models were checked for normality, of which model residuals with a Shapiro-Wilk W value of >0.9 and a skewness and kurtosis between −2 and 2 were considered normally distributed.

Before weaning, average daily creep feed intake (after log transformation) was analyzed in a general linear mixed model with a spatial power covariance structure, a random effect of litter, and fixed main effects of batch (1 vs. 2) and period (d2-15, d15-21, d21-30). The effect of (observation) day on the binary variable “eater” (1=eater, 0=non-eater) was analyzed in a generalized linear mixed model with a binary distribution and logit link function that, apart from a fixed effect of batch and day (d11, d16, d22, d27) and a random effect of litter, also included a random effect of litter nested within day (split-plot) to account for dependence between littermates within a day. To illustrate the increase in “eaters” over time we calculated and reported the percentage of eaters by dividing the number of eaters per litter by the total number of piglets in the same litter at that observation day. Piglet average daily gain (ADG) and BW were analyzed in a general linear mixed model with fixed main effects of batch and creep feed provision (CF vs. NF), a random effect of litter, and litter size as covariate. Uniformity in BW expressed as coefficient of variation (CV) was analyzed in a general linear model with fixed main effects of batch and creep feed provision. Behavior in the farrowing pen was analyzed in a generalized linear mixed model with a binomial distribution, logit link function and an additional multiplicative over-dispersion parameter. The model included fixed effects of batch, and fixed effects and interactions of creep feed provision and day. In addition, the model included a random effect of litter and random effect of litter nested within day (split-plot).

After weaning, piglet ADG and BW were analyzed in a general linear mixed model with fixed effects of batch, and main effects and interactions of pre-weaning creep feed provision (CF vs. NF) and post-weaning creep feed supplementation (CS vs. CON). Moreover, the model included a random effect of weaner pen and litter. Average daily feed intake (ADFI), uniformity in BW expressed as CV, feed conversion ratio (FCR) and mean FCS were analyzed in a general linear model on pen level with the same fixed effects as for post-weaning ADG and BW. CV in BW at one day before weaning was used as covariate in the analyses of CV in BW at 14 days post-weaning. For ADFI data between d0-2 post-weaning no batch effect was included as it was recorded in batch 2 only as result of technical difficulties in batch 1. Average daily intake of the creep feed supplement within CS was analyzed in a general linear model with fixed effects of batch and creep feed provision. The duration of diarrhea (score 3 + 4) was analyzed in a generalized linear model using a Poisson distribution, log link function and an additional multiplicative over-dispersion parameter with the same fixed main effects as used for mean FCS. Behavior in the weaner pen was analyzed in a generalized linear mixed model using a binomial distribution, logit link function and an additional multiplicative overdispersion parameter. The model included the same fixed effects and random effects as mentioned for post-weaning ADG and BW.

Haptoglobin concentrations were analyzed after log transformation in a general linear mixed model with a spatial power covariance structure and a random effect of piglet. The model had fixed main effects of pre-weaning creep feed provision, day, their interactions, as well as batch.

Fixed effects with *P* < 0.05 were further analyzed using *post-hoc* pairwise comparisons of least squares means, with Tukey adjustment for multiple comparisons. Untransformed data are presented as means ± SEM based on pen averages, except for haptoglobin data, which were based on individual piglet data. Log-transformed data are presented as back-transformed LS-means and their 95% CIs.

## Results

### Creep Feed Intake by CF-Litters

In CF-litters, creep feed intake increased with age [*F*_(2, 22)_ = 61.15, *P* < 0.0001, Supplementary Figure S2]. Piglets consumed on average 397 ± 71 g creep feed before weaning (between d2-30), of which 74% was consumed from d21 onwards. Creep feed intake was variable between CF-litters, with cumulative creep feed intake ranging from 96 to 972 g/piglet pre-weaning based on total creep feed consumption per litter.

The percentage of eaters per CF-litter, based on the behavioral observation of eating at least once during scan sampling, increased with age [*F*_(3, 33)_ = 14.80, *P* < 0.0001, Supplementary Figure S2]. The minimum and maximum percentage of eaters per litter was 0 and 69.2% at d11, 0 and 100% at d16, 44.4 and 100% at d22, and 58.3 and 100% at d27.

### Effects of Creep Feed Provision on Piglet Behavior and Performance Before Weaning

**Figure 2:**
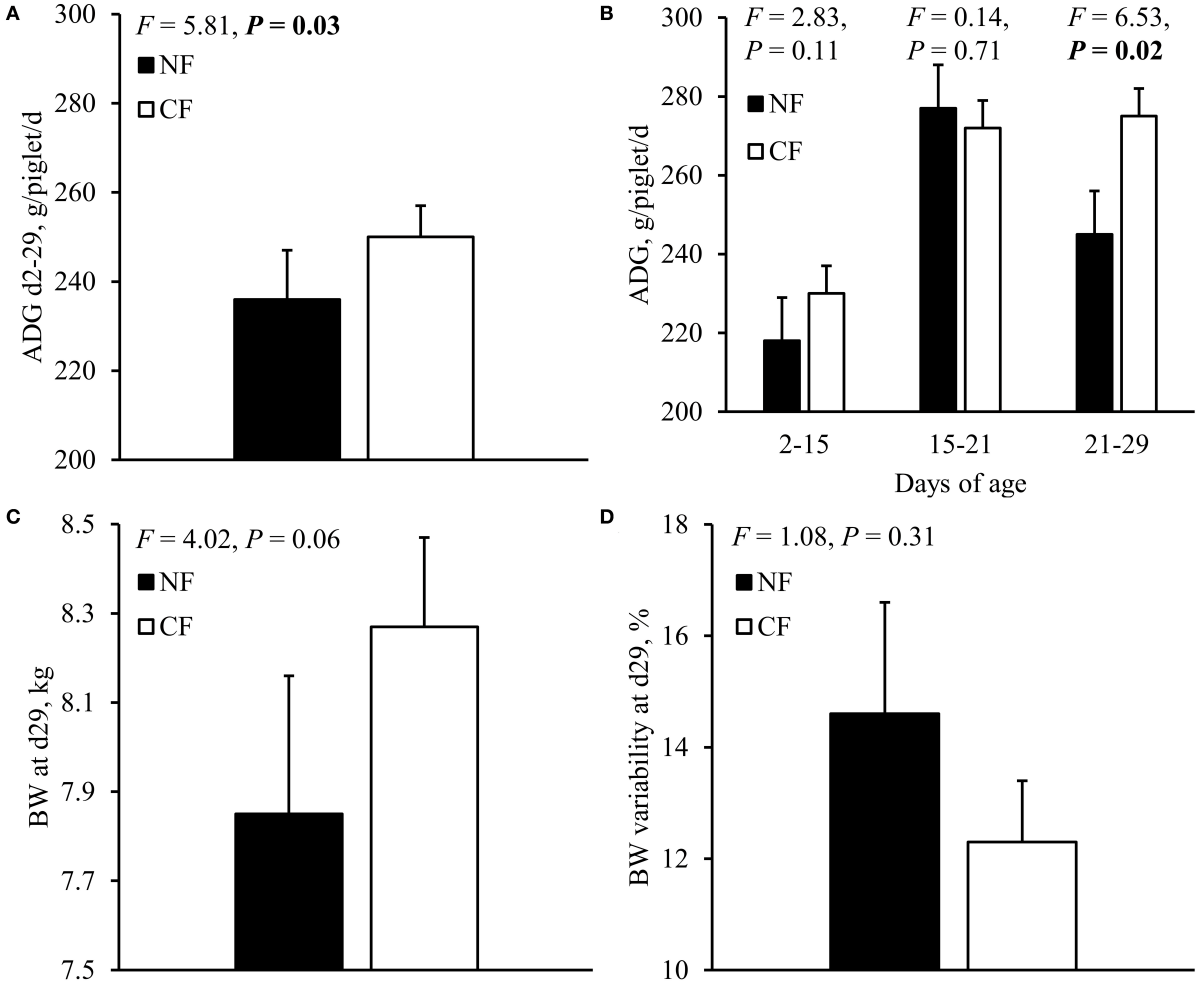
Pre-weaning growth before weaning **(A,B)**, and BW **(C)** and BW variability (**D**, coefficient of variation in BW) at weaning (29 days of age) of piglets that were provided with creep feed from 2 days of age (CF) or not (NF). *P*-values < 0.05 are presented in bold. *F*_(1, 18)_ for pre-weaning growth and BW, and *F*_(1, 19)_ for pre-weaning BW variability.

ADG of CF-piglets throughout lactation (d2-29) was higher than that of NF-piglets (Figure 2A). Analysis per time period revealed that the ADG of CF-piglets did not differ from that of NF-piglets in the first two weeks of lactation and in the third week of lactation, but was increased as compared with NF-piglets in the last week before weaning at 4 weeks of age (Figure 2B). BW at d29 (Figure 2C) and litter uniformity in BW at d29 (Figure 2D) were not affected by the provision of creep feed.

CF-piglets spent 2.9 ± 0.5% of their time either exploring or eating the feed, which were behaviors the NF-piglets could not perform. Time spent on other behaviors in the farrowing room (sucking and massaging udder, drinking, exploring environment, inactive behavior, playing and manipulating pen mates or the sow) were unaffected by providing creep feed (Table 3).

**Table 3:** Effects of creep feed provision from 2 days of age on piglet behavior before weaning (d11, 16, 22 and 27 of age).

**Behavior, % of observations**	**NF**	**CF**	**Significance (*****F*****-value**^a^, ***P*****-value)**		
			**PRE**	**Day**	**PRE x Day**
Suckling and massaging udder	15.6 ± 1.2	13.6 ± 0.7	0.76, 0.40	7.30, **0.0003**	2.41, 0.08
Drinking	0.3 ± 0.1	0.3 ± 0.1	0.29, 0.60	3.20, **0.03**	0.14, 0.93
Eating feed	-	2.6 ± 0.5	-	-	-
Exploring feed	-	0.3 ± 0.04	-	-	-
Exploring environment	14.4 ± 0.8	12.8 ± 1.0	0.91, 0.35	46.61, **<0.0001**	1.22, 0.31
Inactive behavior	54.1 ± 1.8	55.3 ± 2.1	0.05, 0.83	6.22, **0.001**	1.21, 0.31
Playing	2.1 ± 0.2	2.3 ± 0.2	0.07, 0.80	1.19, 0.32	0.17, 0.92
Manipulating pen mates	1.0 ± 0.1	0.9 ± 0.07	0.04, 0.85	0.48, 0.70	0.07, 0.97
Manipulating sow	0.6 ± 0.08	0.4 ± 0.05	2.09, 0.16	3.57, **0.02**	0.31, 0.82

*NF, no creep feed during lactation (114 piglets from 10 litters); CF, ad libitum creep feed from d2 of lactation (143 piglets from 12 litters)*.

*PRE, creep feed yes/no pre-weaning*.

*Data are means ± SEM based on pen averages over all observation days, as no interaction between creep feed provision and day was found*.

*P-values < 0.05 are presented in bold*.

a *F_(1, 20)_ for PRE and F_(3, 59)_ for Day and PRE x Day*.

### Effects of Creep Feed Provision and Creep Feed Supplementation on Piglet Behavior and Performance After Weaning

**Figure 3:**
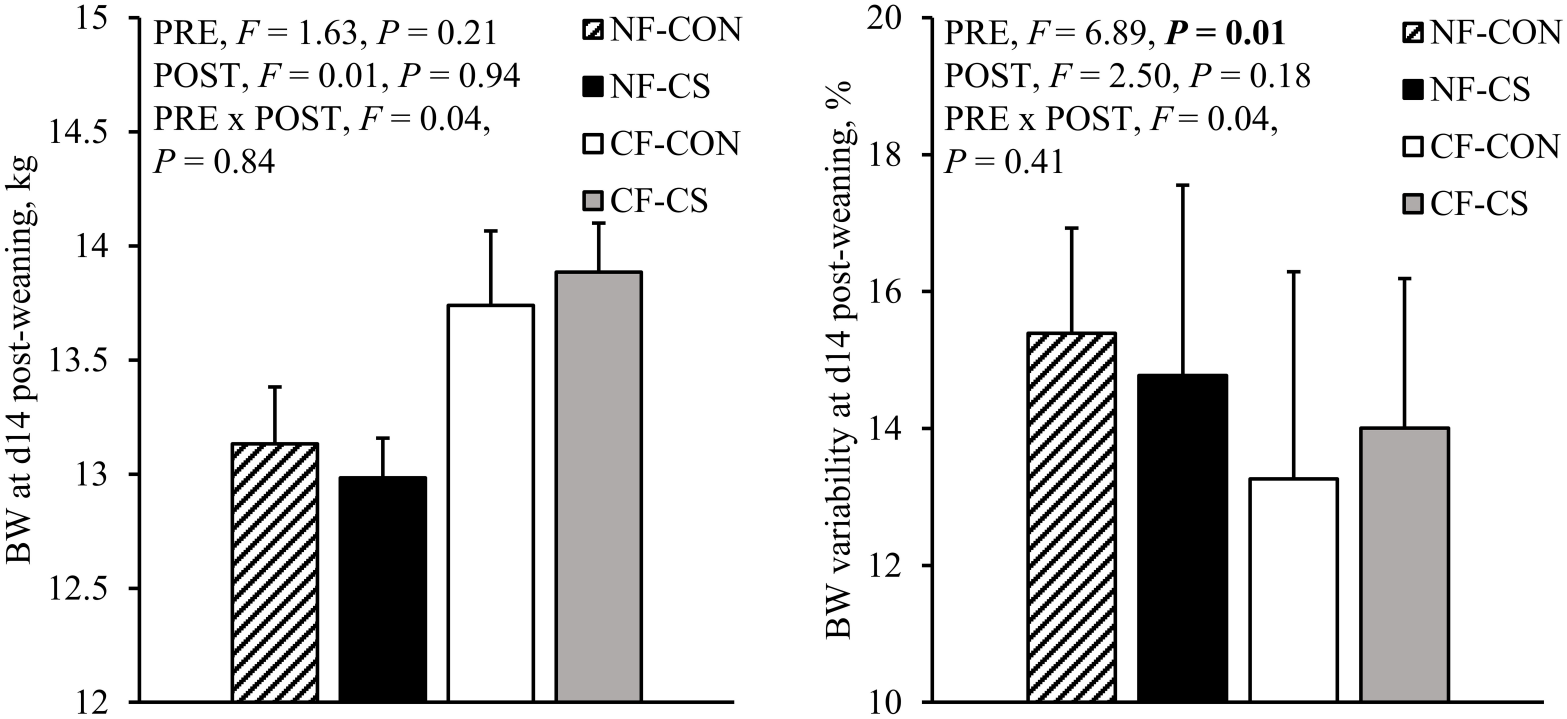
BW and BW variability (coefficient of variation in BW) at 14 days after weaning of piglets that were provided with creep feed from 2 days of age (CF) or not (NF) before weaning and provided with a weaner diet (CON, *n* = 64 piglets in 16 pens) or a creep feed supplement on top of their weaner diet (CS, *n* = 64 piglets in 16 pens) after weaning. PRE = creep feed yes/no pre-weaning. POST = creep feed supplement yes/no post-weaning. Data are means ± SEM based on pen averages. *P*-values < 0.05 are presented in bold. *F*_(1, 27)_ for post-weaning BW and *F*_(1, 26)_ for post-weaning BW variability.

No main effects of pre-weaning creep feed provision, post-weaning creep feed supplementation and their interaction were observed on feed intake or growth during the first two days after weaning (Table 4). However, CF-piglets ate more on the subsequent three days (d2-5 post-weaning, CF: 366 ± 16 vs. NF: 296 ± 12 g/d), which was reflected in a greater ADG of CF-piglets between d2-5 post-weaning compared to NF-piglets (CF: 313 ± 19 vs. NF: 248 ± 15 g/d). In the following days, between d5-9, performance parameters did not differ between the treatment groups. Between d9-14 post-weaning, pre-weaning creep feed provision affected feed intake, with NF-piglets eating more than CF-piglets (NF: 605 ± 16 vs. CF: 550 ± 20 g/d), although this was not reflected in a higher ADG. Also an effect of creep feed supplementation after weaning was found, with a higher feed intake between d9-14 post-weaning for CS-piglets (CS: 609 ± 18 vs. CON: 547 ± 17 g/d). Creep feed supplementation did, however, not affect ADG in this period and did also not affect feed intake or growth in any of the other time periods studied. Treatments did not affect the total feed intake between d2-14 post-weaning, the growth performance of piglets between d-1-14 post-weaning and FCR between d2-14 post-weaning (Table 4). No effects of the treatments were found on BW at d14 post-weaning either (Figure 3). Nevertheless, the BW of CF-piglets at d14 post-weaning was less variable compared to NF-piglets (CV in BW, CF: 13.6 ± 1.9 vs. NF: 15.1 ± 1.5%, Figure 3).

**Table 4:** Post-weaning piglet performance of piglets that were provided with creep feed from 2 days of age (CF) or not (NF) before weaning and provided with a weaner diet (CON, *n* = 64 piglets in 16 pens) or a creep feed supplement on top of their weaner diet (CS, *n* = 64 piglets in 16 pens) post-weaning.

	**NF**		**CF**		**Significance (*****F*****-value**^c^, ***P*****-value)**		
	**CON**	**CS**	**CON**	**CS**	**PRE**	**POST**	**PRE x POST**
ADFI, g/pig/d							
d 0–2^a^	182 ± 20	169 ± 11	127 ± 16	150 ± 26	4.17, 0.06	0.04, 0.84	1.03, 0.33
d 2–5	313 ± 16	279 ± 17	380 ± 23	352 ± 21	12.83, **0.001**	2.54, 0.12	0.03, 0.88
d 5–9	389 ± 13	347 ±13	390 ± 24	396 ± 20	1.82, 0.19	0.97, 0.33	1.62, 0.21
d 9–14	575 ± 22	636 ± 18	519 ± 23	582 ± 30	5.71, **0.02**	7.26, **0.01**	0.00, 0.97
d 2–14	447 ± 6	450 ± 10	441 ± 8	462 ± 9	0.11, 0.75	2.03, 0.17	1.12, 0.30
ADG, g/pig/d							
d−1–2	218 ± 19	175 ± 19	167 ± 21	203 ± 21	0.25, 0.62	0.01, 0.94	3.23, 0.08
d 2–5	236 ± 24	259 ± 19	315 ± 31	310 ± 24	6.49, **0.02**	0.14, 0.72	0.33, 0.57
d 5–9	356 ± 21	339 ± 16	303 ± 22	336 ± 23	0.50, 0.49	0.14, 0.71	0.90, 0.35
d 9–14	488 ± 25	484 ± 21	547 ± 28	532 ± 19	1.93, 0.18	0.20, 0.65	0.30, 0.59
d−1–14	348 ± 13	339 ± 10	359 ± 21	369 ± 12	0.79, 0.38	0.00, 0.96	0.11, 0.74
FCR, d 2–14	1.12 ± 0.08	1.15 ± 0.05	1.14 ± 0.06	1.22 ± 0.03	0.75, 0.39	0.83, 0.37	0.15, 0.70
Mean FCS^b^	0.31 ± 0.06	0.42 ± 0.06	0.31 ± 0.06	0.34 ± 0.07	0.41, 0.53	1.13, 0.30	0.41, 0.53
No. of days with diarrhea	4.38 ± 0.78	5.25 ± 0.56	4.13 ± 0.85	4.25 ± 0.98	0.54, 0.47	0.33, 0.57	0.17, 0.68

*PRE, creep feed yes/no pre-weaning; POST, creep feed supplement yes/no post-weaning*.

*Data are means ± SEM based on pen averages. P < 0.05 are presented in bold*.

a *ADFI between d 0–2 was analyzed for batch 2 only as result of technical difficulties in batch 1*.

b *Fecal consistency score*.

c *F_(1, 27)_ for all parameters except for ADFI between d 0–2, which was F_(1, 12)_*.

Within CS, the creep feed supplement was consumed in greater amounts by NF- than CF-piglets throughout the first two weeks post-weaning (d0-14, NF-CS: 40 ± 0.5 vs. CF-CS: 33 ± 2.0 g/pig/d, *F*_(1, 13)_ = 14.42, *P* = 0.002).

Diarrhea was first observed in the pens at d2 post-weaning and peaked at d6 post-weaning when half of the pens contained diarrheic fecal pools. Thereafter, diarrhea partially recovered but remained present and peaked for a second time at the end of the experiment at d14 post-weaning. Diarrheic fecal pools of score 3 were highly prevalent (97% of the pens), whereas pools of score 4 were less prevalent (25% of the pens). The prevalence of watery diarrhea in the first 2 weeks post-weaning (% of pens with ≥ 1 day watery diarrhea) was 25, 37.5, 0, and 37.5% for CF-CON, CF-CS, NF-CON, and NF-CS, respectively. The severity and duration of diarrhea observed in the first two weeks post-weaning did not differ between the treatment groups (Table 4).

CS-piglets spent more time on exploring feed than CON-piglets at wk 2 post-weaning (CS: 0.29 ± 0.08 vs. CON: 0.11 ± 0.03%, Table 5). No effects of creep feed provision, creep feed supplementation and their interaction were found on the other post-weaning behaviors (drinking, eating feed, exploring environment, inactive behavior, playing and manipulating pen mates).

**Table 5:** Behavioral activities (% of total observations) in the first 2 weeks after weaning (week 1: 36 days of age, week 2: 44 days of age).

**Behavior after weaning**	**NF**		**CF**		**Significance (*****F*****-value**^a^, ***P*****-value)**		
	**CON**	**CS**	**CON**	**CS**	**PRE**	**POST**	**PRE x POST**
**WEEK 1 AFTER WEANING**							
Drinking	1.0 ± 0.2	1.0 ± 0.1	1.2 ± 0.1	1.1 ± 0.2	0.01, 0.94	0.58, 0.45	0.09, 0.77
Eating feed	11.6 ± 0.7	12.7 ± 1.1	10.4 ± 0.9	11.0 ± 1.1	2.30, 0.14	0.70, 0.41	0.05, 0.83
Exploring feed	0.07 ± 0.03	0.23 ± 0.10	0.07 ± 0.03	0.31 ± 0.11	0.00, 0.96	3.18, 0.09	0.75, 0.39
Exploring environment	22.8 ± 2.4	23.8 ± 2.7	24.0 ± 1.0	21.8 ± 1.4	0.01, 0.91	0.12, 0.73	0.65, 0.43
Inactive behavior	46.8 ± 3.1	47.3 ± 3.7	47.4 ± 2.4	50.6 ± 3.4	0.52, 0.48	0.46, 0.50	0.31, 0.58
Playing	3.2 ± 0.6	2.9 ± 0.6	3.3 ± 0.4	2.3 ± 0.3	0.05, 0.82	2.11, 0.16	0.48, 0.49
Manipulating pen mates	1.3 ± 0.3	1.3 ± 0.3	1.3 ± 0.4	1.0 ± 0.3	0.09, 0.77	0.60, 0.45	1.05, 0.31
**WEEK 2 AFTER WEANING**							
Drinking	1.2 ± 0.1	1.2 ± 0.2	1.4 ± 0.2	1.3 ± 0.2	0.93, 0.34	0.03, 0.86	0.00, 0.98
Eating feed	10.4 ± 1.1	10.5 ± 0.7	10.9 ± 1.0	11.5 ± 0.6	0.64, 0.43	0.15, 0.70	0.09, 0.76
Exploring feed	0.07 ± 0.03	0.31 ± 0.14	0.16 ± 0.05	0.26 ± 0.08	0.81, 0.38	5.27, **0.03**	1.02, 0.32
Exploring environment	33.4 ± 1.5	28.4 ± 3.0	29.5 ± 1.9	26.8 ±1.7	1.57, 0.22	3.43, 0.07	0.28, 0.60
Inactive behavior	39.9 ± 1.9	45.7 ± 3.4	43.0 ± 2.7	45.5 ± 2.5	0.27, 0.61	2.36, 0.14	0.37, 0.55
Playing	2.8 ± 0.5	2.7 ± 0.4	3.2 ± 0.4	2.5 ± 0.4	0.02, 0.88	0.71, 0.41	0.58, 0.45
Manipulating pen mates	1.7 ± 0.5	1.7 ± 0.7	1.8 ± 0.5	1.9 ± 0.4	0.45, 0.51	0.02, 0.90	0.04, 0.85

*Piglets were provided with creep feed from 2 days of age (CF) or not (NF) before weaning and provided with a weaner diet (CON, n = 64 piglets in 16 pens) or a creep feed supplement on top of their weaner diet (CS, n = 64 piglets in 16 pens) after weaning. PRE, creep feed yes/no pre-weaning; POST, creep feed supplement yes/no post-weaning. Data are means ± SEM based on pen averages*.

*P < 0.05 are presented in bold*.

a *F_(1, 27)_ for all parameters*.

### Effects of Creep Feed Provision on Plasma Haptoglobin Concentrations Before and After Weaning

**Figure 4:**
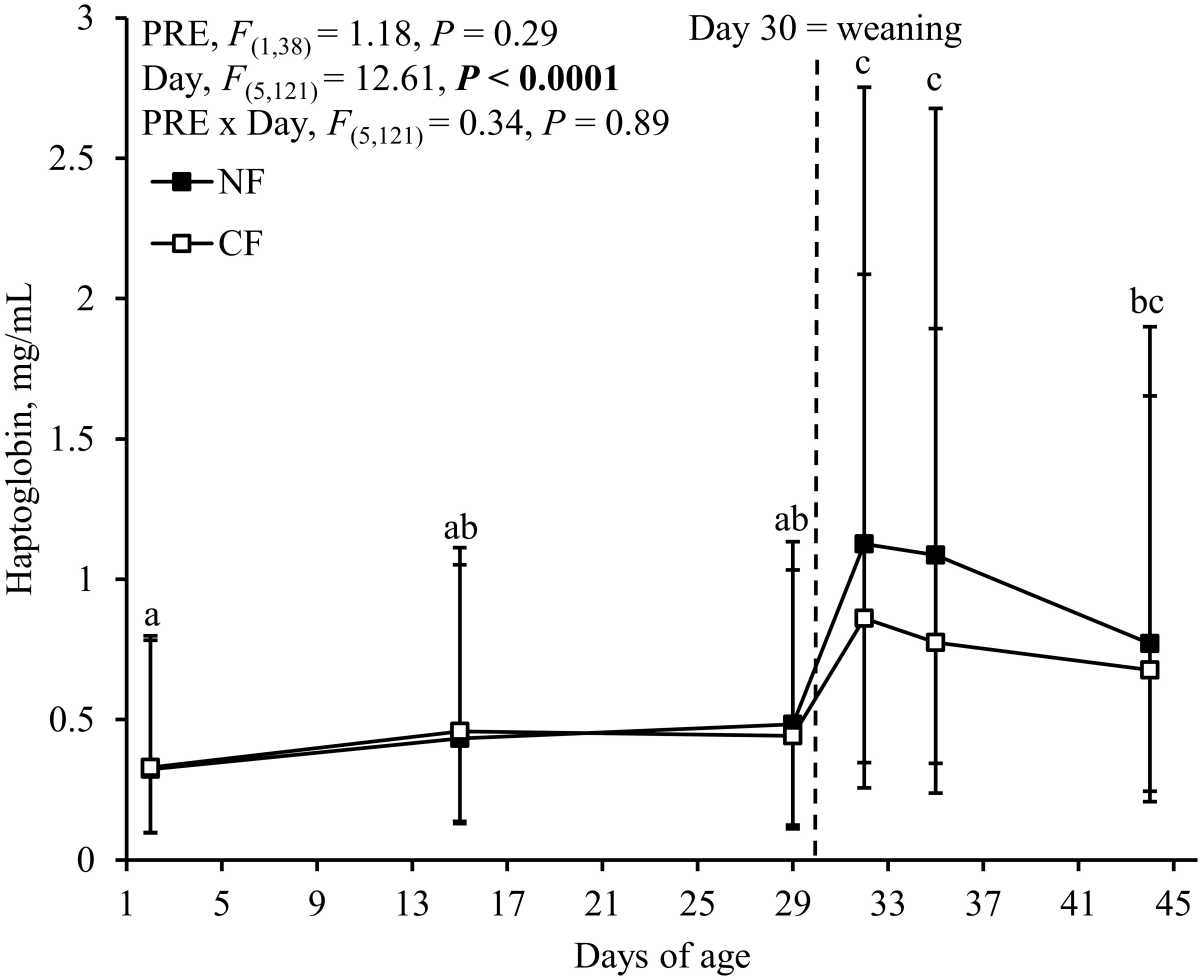
Plasma haptoglobin concentrations of piglets that were provided with creep feed from 2 days of age (CF) or not (NF) before weaning and provided with a weaner diet after weaning. PRE = creep feed yes/no pre-weaning. Haptoglobin was determined at d29 after birth in 21 CF- and 20 NF-piglets. In 28 of these piglets additional samples were taken before weaning (*n* = 14 per treatment) and/or after weaning (*n* = 13 CF- and 12 NF-piglets). Data are back-transformed LS-means and their 95% CIs. Superscripts without a common letter differ over time at *P* < 0.05. *P*-values < 0.05 are presented in bold.

There was no difference in absolute haptoglobin concentrations between NF- and CF-piglets for any of the time points measured (Figure 4). Moreover, creep feed provision did not affect the difference in haptoglobin concentrations between two successive time points (data not shown). Irrespective of treatment however, the concentration of haptoglobin was affected by day and peaked at d2 after weaning (Figure 4). Absolute values of haptoglobin at d2 and d5 after weaning were greater than pre-weaning values (*post-hoc* comparisons, *P* ≤ 0.002 for all). In addition, the concentration of haptoglobin at d2 after birth was lower than the concentration of haptoglobin at d14 post-weaning (*post-hoc* comparison, *P* = 0.0005).

## Discussion

The present study was performed to determine the impact of pre-weaning creep feed provision, post-weaning creep feed supplementation and their interaction on piglet behavior and performance after weaning. We hypothesized that piglets with access to the creep feed both before weaning and as a supplement after weaning would experience the lowest level of food neophobia compared to the other treatment groups and therefore ingest the largest amount of feed and perform the best in the first days after weaning. The results of this study did not confirm this hypothesis. Effects of pre-weaning creep feed provision and post-weaning creep feed supplementation will be discussed separately below.

### Effects of Pre-weaning Creep Feed Provision

Given that piglets provided with creep feed would be more familiarized with solid feed before weaning, we anticipated them to display increased feed intake after weaning. As a result, we hypothesized that creep feed provision would stimulate growth of weaned piglets, and reduce haptoglobin concentrations and weaning-stress-associated behaviors. However, this study did not support this hypothesis. There may be several reasons for this lack of an effect of creep feed provision. Firstly, the housing conditions of the piglets in this study may have been more favorable in terms of behavioral needs compared to commercial farming, as the density of piglets per pen and the number of piglets that shared one chew object simultaneously (2 piglets/chew object) were considerably lower. Hence, when kept in commercial farming conditions, behaviors like chewing and rooting the environment may become more re-directed at pen mates compared to our experiment, where the chewing behavior mainly targeted the environment and chew objects in it. Secondly, stress from removal of the sow (social stress), mixing with unfamiliar piglets (social stress) and housing in a novel pen (environmental stress), may have overruled the stress from the abrupt change in diet (nutritional stress). Hötzel et al. (34) suggested that an increase in aggression, exploratory behavior, activity and vocalizations seemed mainly associated with the environmental and social stressors of weaning, rather than the nutritional stressor. However, the environmental and social stressors were tested in combination with the nutritional stressor and an unweaned control group without any stressors was missing. The contribution of social, environmental and nutritional stress to the stress response at weaning therefore remains unknown and is of interest for future research. Only 3% of the piglets within the sample population had haptoglobin concentrations within the acute range of 3–8 mg/mL (PHASE™ Haptoglobin Assay, Tridelta Development Limited, Maynooth, Ireland), suggesting acute infection or inflammation were likely absent in the majority of the piglets. The observed elevation in haptoglobin after weaning may therefore reflect an increase in stress associated with weaning (8, 9), although there was no control of unweaned piglets in the present study. Lastly, even though provision of creep feed at litter level did not lead to an improved post-weaning feed intake and reduced levels of stress at weaning, this does not exclude a potential favorable effect of creep feed intake in the individual piglets that did ingest the creep feed. Creep feed intake varies substantially, both between litters and littermates, and part of the piglets provided with creep feed in our study were non-eaters according to the scan sampling observations, which may have masked the effects. In support of this, post-weaning performance benefits on feed intake and growth seem more pronounced in piglets with proven intake of creep feed [eaters vs. non-eaters; (2, 18)] and, in particular, in piglets with a high creep feed consumption level [good vs. moderate vs. bad vs. non-eaters; (3, 35)]. Due to the large variation in creep feed intake it is difficult to exactly pinpoint the effects of creep feed provision. Therefore, the relationship between individual creep feed consumption and piglet behavior as well as weaning-induced stress warrants further investigation.

We did not observe differences in overall ADFI, ADG and FCR in the first two weeks post-weaning, or in BW at 14 days post-weaning, between NF- and CF-piglets in line with findings of others (14, 18). However, as in other studies (14, 17), effects of creep feed provision were observed in shorter post-weaning time periods, suggesting that creep feed provision had consequences for the dynamics in post-weaning performance. CF-piglets ate and grew more from d2-5 post-weaning, indicating a faster recovery in energy intake. Although NF-piglets seemed to “catch up” by consuming more between d9-14 post-weaning, this was not reflected by a higher growth than CF-piglets. Furthermore, CF-piglets were more uniform in BW at 14 days post-weaning compared to NF-piglets, but effects of creep feed provision on within-pen variation in post-weaning BW was not studied before. Taken together, we therefore conclude, also given the high number of response parameters tested, that creep feed provision does not have a large impact on post-weaning feed intake or body weight development. The impact of creep feed provision on post-weaning performance found in other studies is inconsistent [positive effects: (14, 16), negative effects: (17), lack of effect: (18, 19)]. The dynamics in post-weaning performance observed as result of creep feed provision, as found in the current study, may partly explain the controversy in results between studies that measured performance at different time points (14, 20). However, inconsistencies regarding the effects of creep feed provision on post-weaning piglet performance are also reported between studies that measured performance in similar time periods. For example, Shea et al. (16) reported a higher BW at 14 days post-weaning as result of creep feed provision, which conflicts with the result in our study. Furthermore, a lower ADG and FCR on one hand (17), but a greater ADFI and ADG on the other hand (16) were reported for CF- vs. NF-piglets in the first two weeks post-weaning. Factors that likely contribute to these inconsistencies include weaning age (36, 37), the duration of creep feed provision (26), the composition of the pre- and post-weaning diet as well as their interaction (38), the percentage of eaters (2) and the intake of creep feed [high vs. low intake per piglet; (3)]. Except for post-weaning diet composition, the other factors may also explain the occurrence of both positive effects (16, 20, 39) as well as no effects [e.g. (14, 18, 19)] of creep feed provision on pre-weaning ADG and weaning weight. Lastly, we hypothesized that CF-piglets would be more explorative than NF-piglets due to the provision of fibrous creep feed. Creep feed provision did, however, not affect the behavior of piglets in the farrowing pen.

### Effects of Post-weaning Creep Feed Supplementation

The creep feed supplement was consumed in greater amounts by NF- than CF-piglets after weaning, while we expected the opposite due to reduced neophobia of CF-piglets toward this feed. We think a novelty effect would not be the sole explanation, as the effect lasted for the first two weeks after weaning. The larger pellet sizes in the creep feed supplement may facilitate handling of the pellets by NF-piglets (22), which possibly have less mature motoric jaw movements. This would be in line with studies in which an increased post-weaning feed intake of a large diameter pellet was reported for piglets that were relatively inexperienced with solid feed before weaning (40, 41), but not in piglets that were, similar to our CF-piglets, more experienced with solid feed pre-weaning (22, 42). Also dietary variety might mainly help inexperienced piglets to start eating solid feed by stimulating exploration toward the feed and therefore intake (23, 43).

We predicted post-weaning supplementation of creep feed to increase feed exploration and intake and to reduce weaning-stress-induced behaviors in the weaner pen by reducing food neophobia of piglets that were given creep feed before weaning. Indeed, creep feed supplementation after weaning increased the time spent on exploratory behavior toward the feed, both in CF- and in NF-piglets at week 2 post-weaning. This might be the result of dietary variety established by the two feeds provided [as suggested by (23, 44)] or of piglets selecting their preferred (size of the) feed item. More time on feed exploration may also result from offering larger pellet sizes, which are better suited for (playful) exploration [as suggested by (22, 42)], within the creep feed supplement. Creep feed supplementation also improved feed intake, but only between d9-14 post-weaning. As we expect piglets to be experienced with solid feed by then, we hypothesize that this increase in feed intake was mainly related to dietary variety (23, 43) rather than the larger pellet sizes in the creep feed supplement (22, 42). The observed increase in feed intake between d9-14 post-weaning was due to a higher intake of the creep feed supplement as well as a numerically greater intake of the weaner diet. Overall ADFI, ADG, FCR and fecal consistency scores in the first two weeks post-weaning did not differ between creep-supplemented piglets and control piglets that did not receive creep feed on top of their weaner diet. Post-weaning creep feed supplementation also did not affect behaviors other than feed exploration.

Post-weaning creep feed supplementation thus showed positive effects on feed exploration and intake in the second week after weaning, although the creep feed was only provided in a limited amount on top of the weaner diet. This might have been the result of dietary variety established by the two feeds (23, 43), which were the creep feed and weaner diet. We recommend future research to study the effect of dietary variety, for example by offering two feeds *ad libitum* and simultaneously, on the behavior and performance of weaner piglets.

## Conclusions

Results of the present study indicate that providing piglets fibrous creep feed before weaning and as a supplement on top of their weaner diet after weaning had no clear effects on piglet behavior and performance. The results therefore do not support that creep feed provision may reduce the nutritional stressor at weaning.

## Data Availability Statement

All datasets generated for this study are included in the article/Supplementary Material.

## Ethics Statement

The animal study was reviewed and approved by the Animal Care and Use committee of Wageningen University & Research (Wageningen, The Netherlands).

## Author Contributions

All authors designed the experiment. AM and RC conducted the experiment. AM analyzed the data, wrote the manuscript and prepared the figures. JB advised on data analyses. RC, WG, BK, MK, and JB substantively revised the manuscript. All authors approved the submitted version.

## Conflict of Interest

The study was co-financed by Cargill Animal Nutrition and Coppens Diervoeding. The authors declare that the research was conducted in the absence of any commercial or financial relationships that could be construed as a potential conflict of interest.

## References

[1] Weary DM Jasper J Hötzel MJ Understanding weaning distress. Appl Anim Behav Sci. (2008) 110:24–41. 10.1016/j.applanim.2007.03.025

[2] Bruininx EM Binnendijk GP van der Peet-Schwering CMC Schrama JW den Hartog LA Everts H. Effect of creep feed consumption on individual feed intake characteristics and performance of group-housed weanling pigs. J Anim Sci. (2002) 80:1413–8. 10.2527/2002.8061413x 12078720

[3] Bruininx EM Schellingerhout AM Binnendijk AB van der Peet-Schwering GP Schrama CMC den Hartog JW Individually assessed creep feed consumption by suckled piglets: influence on post-weaning food intake characteristics and indicators of gut structure and hind-gut fermentation. Anim Sci. (2004) 78:67–75. 10.1017/S1357729800053856

[4] Heo JM Opapeju FO Pluske JR Kim JC Hampson DJ Nyachoti CM. Gastrointestinal health and function in weaned pigs: a review of feeding strategies to control post-weaning aa without using in-feed antimicrobial compounds. J Anim Physiol Anim Nutr. (2013) 97:207–37. 10.1111/j.1439-0396.2012.01284.x 22416941

[5] Gresse R Chaucheyras-Durand F Fleury MA van de Wiele T Forano E Blanquet-Diot S. Gut microbiota dysbiosis in postweaning piglets: understanding the keys to health. Trends Microbiol. (2017) 25:851–73. 10.1016/j.tim.2017.05.004 28602521

[6] Colson V Orgeur P Foury A Mormède P Consequences of weaning piglets at 21 and 28 days on growth, behaviour and hormonal responses. Appl Anim Behav Sci. (2006) 98:70–88. 10.1016/j.applanim.2005.08.014

[7] Van Nieuwamerongen SE Soede NM van der Peet-Schwering CMC Kemp B Bolhuis JE Gradual weaning during an extended lactation period improves performance and behavior of pigs raised in a multi-suckling system. Appl Anim Behav Sci. (2017) 194:24–35. 10.1016/j.applanim.2017.05.005

[8] Pomorska-Mól M Kwit K Markowska-Daniel I Major acute phase proteins in pig serum from birth to slaughter. Bull Vet Inst Pulawy. (2012) 56:553–7. 10.2478/v10213-012-0097-y

[9] De UK Nandi S Mukherjee R Gaur GK Verma MR Identification of some plasma biomarkers associated with early weaning stress in crossbred piglets. Comp Clin Pathol. (2017) 26:343–9. 10.1007/s00580-016-2379-x

[10] Piñeiro C Piñeiro M Morales J Carpintero R Campbell FM Eckersall PD. Pig acute-phase protein levels after stress induced by changes in the pattern of food administration. Animal. (2007) 1:133–9. 10.1017/S1751731107283909 22444216

[11] Pastorelli H Le Floc'h N Merlot E Meunier-Salaün MC van Milgen J Montagne L. Feed restriction applied after weaning has different effects on pig performance and health depending on the sanitary conditions. J Anim Sci. (2012) 90:4866–75. 10.2527/jas.2012-5309 22952368

[12] Hiss S Sauerwein H. Influence of dietary ß-glucan on growth performance, lymphocyte proliferation, specific immune response and haptoglobin plasma concentrations in pigs. J Anim Physiol Anim Nutr. (2003) 87:2–11. 10.1046/j.1439-0396.2003.00376.x 14511144

[13] Bolhuis JE Oostindjer M van den Brand H Gerrits WJJ Kemp B Voluntary feed intake in piglets: potential impact of early experience with flavours derived from the maternal diet. In: Torrallardona D. Roura E. editors, Voluntary Feed Intake in Pigs. Wageningen Pers, Wageningen: Netherlands (2009). p. 37–61.

[14] Muns R Magowan E. The effect of creep feed intake and starter diet allowance on piglets' gut structure and growth performance after weaning. J Anim Sci. (2018) 96:3815–23. 10.1093/jas/sky239 29924319 PMC6127789

[15] Figueroa J Marchant I Morales P Salazar LC. Do prenatally-conditioned flavor preferences affect consumption of creep feed by piglets? Animals. (2019) 9:944. 10.3390/ani9110944 31717648 PMC6912572

[16] Shea JN Beaulieu AD Gillis DA Brown J Creep feeding in the farrowing room: do the outcomes depend on weaning age? In: 2012-2013 Annual Research Report Prairie Swine Centre. Univ. of Saskatchewan, Saskatoon (2012). p. 35–7.

[17] Beaulieu AD Shea J Gillis D Weaning at 28 days. Is creep feeding beneficial? In: 2010 Annual Research Report Prairie Swine Centre. Univ. of Saskatchewan, Saskatoon (2010). p. 49–51.

[18] Sulabo RC Jacela JY Tokach MD Dritz SS Goodband RD DeRouchey JM. Effects of lactation feed intake and creep feeding on sow and piglet performance. J Anim Sci. (2010) 88:3145–53. 10.2527/jas.2009-2131 20495122

[19] Park BC Ha DM Park MJ Lee CY. Effects of milk replacer and starter diet provided as creep feed for suckling pigs on pre- and post-weaning growth. Anim Sci J. (2014) 85:872–8. 10.1111/asj.12246 25039284

[20] Van der Meulen J Koopmans SJ Dekker RA Hoogendoorn A. Increasing weaning age of piglets from 4 to 7 weeks reduces stress, increases post-weaning feed intake but does not improve intestinal functionality. Animal. (2010) 4:1653–61. 10.1017/S1751731110001011 22445118

[21] Kuller WI Tobias TJ van Nes A Creep feed intake in unweaned piglets is increased by exploration stimulating feeder. Livest Sci. (2010) 129:228–31. 10.1016/j.livsci.2010.01.003

[22] Van den Brand H Wamsteeker D Oostindjer M van Enckevort LCM van der Poel AFB Kemp B. Effects of pellet diameter during and after lactation on feed intake of piglets pre- and post-weaning. J Anim Sci. (2014) 92:4145–53. 10.2527/jas.2014-7408 25185217

[23] Middelkoop A Choudhury R Gerrits WJJ Kemp B Kleerebezem M Bolhuis JE Dietary diversity affects feeding behaviour of scukling piglets. Appl Anim Behav Sci. (2018) 205:151–8. 10.1016/j.applanim.2018.05.006

[24] Fleming SA Monaikul S Patsavas AJ Waworuntu RV Berg BM Dilger NR. Dietary polydextrose and galactooligosaccharide increase exploratory behavior, improve recognition memory, and alter neurochemistry in the young pig. Nutr Neurosci. (2017) 22:1–14. 10.1080/1028415X.2017.1415280 29251222

[25] Clouard C Stokvis L Bolhuis JE van Hees HMJ. Short communication: Insoluble fibres in supplemental pre-weaning diets affect behaviour of suckling piglets. Animal. (2018) 12:329–33. 10.1017/S1751731117001501 28701236

[26] Yan L Jang HD Kim IH Effects of varying creep feed duration on pre-weaning and post-weaning performance and behavior of piglet and sow. J Anim Sci. (2011) 24:1601–6. 10.5713/ajas.2011.11181

[27] Van Hees HMJ Davids M Maes D Millet S Possemiers S den Hartog LA. Dietary fibre enrichment of supplemental feed modulates the development of the intestinal tract in suckling piglets. J Animal Sci Biotechnol. (2019) 10:83. 10.1186/s40104-019-0386-x 31636904 PMC6794736

[28] Heo PS Kim DH Jang JC Hong JS Kim YY. Effects of different creep feed types on pre-weaning and postweaning performance and gut development. Asian-Australasian J Anim Sci. (2018) 31:1956–62. 10.5713/ajas.17.0844 30380814 PMC6212741

[29] CVB Veevoedertabel 2007: chemische samenstellingen en nutritionele waarden van voedermiddelen. Centraal Veevoederbureau, Den Haag, The Netherlands (2007).

[30] Oostindjer M Bolhuis JE Mendl M Held S Gerrits WJ van den Brand H. Effects of environmental enrichment and loose housing of lactating sows on piglet behaviour before and after weaning. Appl Anim Behav Sci. (2011) 134:31–41. 10.1016/j.applanim.2011.06.011 20622185

[31] Oostindjer M Mas-Muñoz J van den Brand H Kemp B Bolhuis JE. Maternal presence and environmental enrichment affect food neophobia of piglets. Biol Lett. (2011) 7:19–22. 10.1098/rsbl.2010.0430 20554557 PMC3030865

[32] Pedersen KS Toft N. Intra- and inter-observer agreement when using a descriptive classification scale for clinical assessment of faecal consistency in growing pigs. Prev Vet Med. (2011) 98:288–91. 10.1016/j.prevetmed.2010.11.016 21185096

[33] Christoffersen BØ Jensen SJ Ludvigsen TP Nilsson SK Grossi AB Heegaard PMH. Age- and sex-associated effects on acute-phase proteins in Göttingen minipigs. Comp Med. (2015) 65:333–41. 26310463 PMC4549679

[34] Hötzel MJ de Souza GPP Dalla Costa OA Machado Filho LCP Disentangling the effects of weaning stressors on piglets' behaviour and feed intake: Changing the housing and social environment. Appl Anim Behav Sci. (2011) 135:44–50. 10.1016/j.applanim.2011.09.003

[35] Pluske JR Kim JC Hansen CF Mullan BP Payne HG Hampson DJ. Piglet growth before and after weaning in relation to a qualitative estimate of solid (creep) feed intake during lactation: a pilot study. Arch Anim Nutr. (2007) 61:469–80. 10.1080/17450390701664249 18069618

[36] Callesen J Halas D Thorup F Bach Knudsen KE Kim JC Mullan BP The effects of weaning age, diet composition, and categorization of creep feed intake by piglets on diarrhea and performance after weaning. Livest Sci. (2007) 108:120–3. 10.1016/j.livsci.2007.01.014

[37] Collins CL Morrison RS Smits RJ Henman DJ Dunshea FR Pluske JR Interactions between piglet weaning age and dietary creep feed composition on lifetime growth performance. Anim Prod Sci. (2013) 53:1025–32. 10.1071/AN12009

[38] Torrallardona D Andrés-Elias N López-Soria S Badiola I Cerdà-Cuéllar M Effect of feeding different cereal-based diets on the performance and gut health of weaned piglets with or without previous access to creep feed during lactation. J Anim Sci. (2012) 90:31–3. 10.2527/jas.53912 23365275

[39] Lee SI Kim IH Creep feeding improves growth performance of suckling piglets. R Bras Zootec. (2018) 47:e20170081 10.1590/rbz4720170081

[40] Traylor SL Behnke KC Hancock JD Sorrell P Hines RH Effect of pellet size on growth performance in nursery and finishing pigs. J Anim Sci. (1996) 74:67.

[41] Clark AB de Jong JA DeRouchey JM Tokach MD Dritz SS Goodband RD Effects of creep feed pellet diameter on suckling and nursery pig performance. J Anim Sci. (2016) 94:100–1. 10.2527/msasas2016-213

[42] Edge HL Dalby JA Rowlinson P Varley MA The effect of pellet diameter on the performance of young pigs. Livest Prod Sci. (2005) 97:203–9. 10.1016/j.livprodsci.2005.04.009

[43] Middelkoop A van Marwijk MA Kemp B Bolhuis JE. Pigs like it varied; Feeding behavior and pre- and postweaning performance of piglets exposed to dietary diversity and feed hidden in substrate during lactation. Front Vet Sci. (2019) 6:408. 10.3389/fvets.2019.00408 31803769 PMC6877737

[44] Adeleye OO Guy JH Edwards SA Exploratory behaviour and performance of piglets fed novel flavoured creep in two housing systems. Anim Feed Sci Technol. (2014) 191:91–7. 10.1016/j.anifeedsci.2014.02.001

