# Supplementary material for: Effects of Creep Feed Provision on Behavior and Performance of Piglets Around Weaning
Source: Front Vet Sci. 2020 Nov 12;7:520035. doi: 10.3389/fvets.2020.520035 (PMC7689248; doi:10.3389/fvets.2020.520035)

Supplementary Material

**Supplementary Table S1.** Distribution of treatments before weaning

| **Batch** | **Farrowing room** | **Farrowing pen** | **Block** | **Treatment** |
| --- | --- | --- | --- | --- |
| 1 | 14 | 14.1 | 1 | Empty |
| 1 | 14 | 14.2 | 1 | NF |
| 1 | 14 | 14.3 | 1 | Reserve |
| 1 | 14 | 14.4 | 1 | CF |
| 1 | 14 | 14.5 | 2 | CF |
| 1 | 14 | 14.6 | 2 | NF |
| 1 | 14 | 14.7 | 2 | CF |
| 1 | 14 | 14.8 | 2 | Empty |
| 1 | 15 | 15.1 | 1 | Empty |
| 1 | 15 | 15.2 | 1 | CF |
| 1 | 15 | 15.3 | 1 | NF |
| 1 | 15 | 15.4 | 1 | CF |
| 1 | 15 | 15.5 | 2 | CF |
| 1 | 15 | 15.6 | 2 | NF |
| 1 | 15 | 15.7 | 2 | Reserve |
| 1 | 15 | 15.8 | 2 | Empty |
| 2 | 14 | 14.1 | 1 | Reserve |
| 2 | 14 | 14.2 | 1 | CF |
| 2 | 14 | 14.3 | 1 | CF |
| 2 | 14 | 14.4 | 1 | NF |
| 2 | 14 | 14.5 | 2 | CF |
| 2 | 14 | 14.6 | 2 | CF |
| 2 | 14 | 14.7 | 2 | Reserve |
| 2 | 14 | 14.8 | 2 | NF |
| 2 | 15 | 15.1 | 1 | Reserve |
| 2 | 15 | 15.2 | 1 | NF |
| 2 | 15 | 15.3 | 1 | CF |
| 2 | 15 | 15.4 | 1 | NF |
| 2 | 15 | 15.5 | 2 | CF |
| 2 | 15 | 15.6 | 2 | NF |
| 2 | 15 | 15.7 | 2 | NF |
| 2 | 15 | 15.8 | 2 | Reserve |

**Supplementary Table S2.** Distribution of treatments after weaning

| **Batch** | **Weaner room** | **Weaner pen** | **Block** | **Treatment** |
| --- | --- | --- | --- | --- |
| 1 | 8 | 8.1 | 1 | CF-CON |
| 1 | 8 | 8.2 | 1 | NF-CS |
| 1 | 8 | 8.3 | 1 | CF-CS |
| 1 | 8 | 8.4 | 1 | NF-CON |
| 1 | 8 | 8.5 | 2 | NF-CS |
| 1 | 8 | 8.6 | 2 | CF-CON |
| 1 | 8 | 8.7 | 2 | CF-CS |
| 1 | 8 | 8.8 | 2 | NF-CON |
| 1 | 9 | 9.1 | 1 | NF-CS |
| 1 | 9 | 9.2 | 1 | CF-CON |
| 1 | 9 | 9.3 | 1 | NF-CON |
| 1 | 9 | 9.4 | 1 | CF-CS |
| 1 | 9 | 9.5 | 2 | NF-CS |
| 1 | 9 | 9.6 | 2 | NF-CON |
| 1 | 9 | 9.7 | 2 | CF-CON |
| 1 | 9 | 9.8 | 2 | CF-CS |
| 2 | 8 | 8.1 | 1 | NF-CS |
| 2 | 8 | 8.2 | 1 | CF-CON |
| 2 | 8 | 8.3 | 1 | NF-CON |
| 2 | 8 | 8.4 | 1 | CF-CS |
| 2 | 8 | 8.5 | 2 | CF-CON |
| 2 | 8 | 8.6 | 2 | NF-CS |
| 2 | 8 | 8.7 | 2 | NF-CON |
| 2 | 8 | 8.8 | 2 | CF-CS |
| 2 | 9 | 9.1 | 1 | CF-CON |
| 2 | 9 | 9.2 | 1 | NF-CS |
| 2 | 9 | 9.3 | 1 | CF-CS |
| 2 | 9 | 9.4 | 1 | NF-CON |
| 2 | 9 | 9.5 | 2 | CF-CS |
| 2 | 9 | 9.6 | 2 | CF-CON |
| 2 | 9 | 9.7 | 2 | NF-CS |
| 2 | 9 | 9.8 | 2 | NF-CON |

**Supplementary Figure S1.** Timeline of all measurements performed on piglets that were provided with creep feed from 2 days of age (CF) or not (NF) before weaning and provided with weaner diet (CON) or a creep feed supplement on top of their weaner diet (CS) post-weaning.


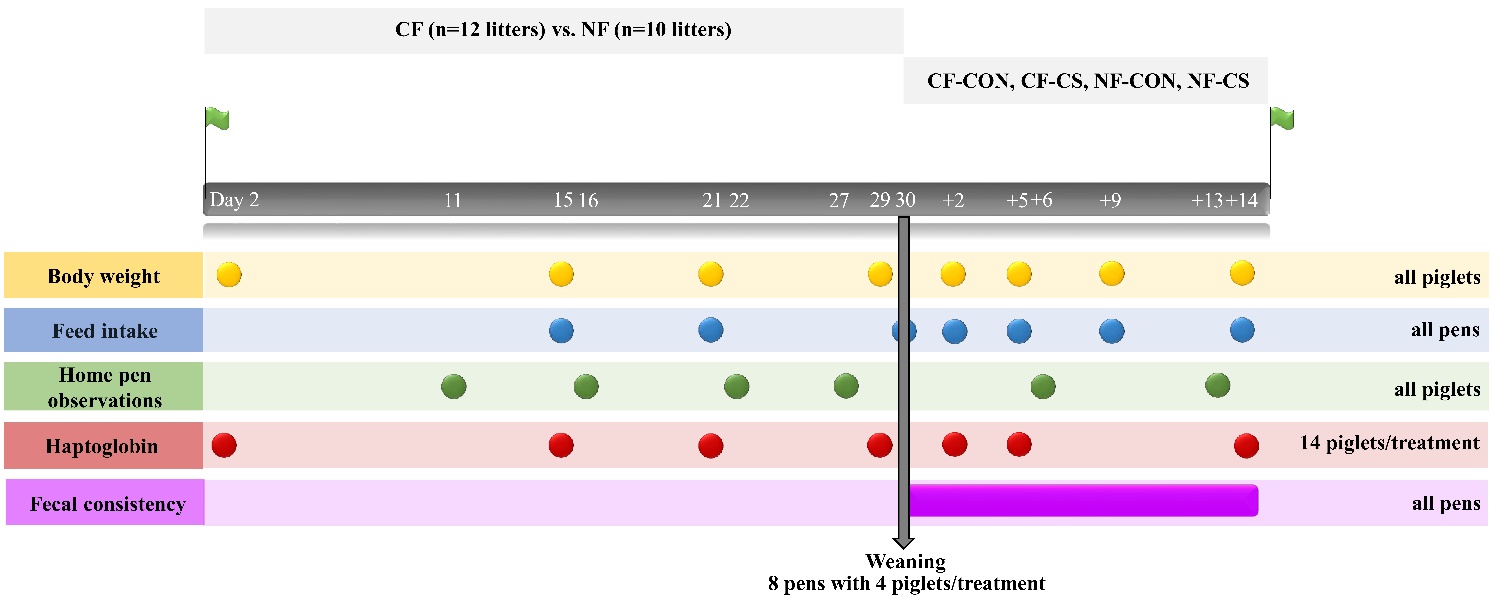


**Supplementary Table S3.** Behavior of piglets before and after weaning

| **Behavior** | | **Description** |
| --- | --- | --- |
| Suckling^1^ | | Drinking milk from teat of sow by sucking movements (soft suckling noises). Milk release by the sow often goes together with pulsating sounds made by the sow and the majority of piglets suckling. |
| Massaging udder^1^ | | Rubbing the udder of the sow by up and down movements of the head/snout |
| Drinking | | Drinking water from drinking trough/nipple by sucking movements |
| “Eating feed” | |  |
|  | Eating feed | Eating or chewing feed (at the feeder) |
|  | Eating feed from floor | Eating or chewing feed from the floor |
| “Exploring feed” | | |
|  | Exploring feed | Sniffing, touching (with snout) or rooting the feed in the feeder |
|  | Exploring feed on floor | Sniffing, touching (with snout) feed on floor |
|  | Playing with feed | Rolling feed item over floor, walking around with feed item in mouth, (energetically) shaking head with feed item in mouth |
| “Exploring environment” | |  |
|  | Nosing environment | Sniffing, touching (with snout) or licking part of the pen (e.g. floor, wall) |
|  | Rooting environment | Rooting part of the pen, scraping floor with one the front legs |
|  | Chewing environment | Chewing or nibbling part of the pen, including chew object |
|  | Chewing feces or sham chewing | Chewing feces or sham chewing (not chewing on part of the pen, feed or chew object) |
|  | Exploring feeder | Sniffing, touching (with snout), rooting or chewing on feeder |
|  | Exploring drinker | Sniffing, touching (with snout) or chewing on drinking trough |
| “Inactive behavior” | | |
|  | Lying eyes closed | Lying on side or belly with eyes closed without performing any other described behavior |
|  | Lying eyes open | Lying on side or belly with eyes open, sitting or kneeling without performing any other described behavior |
| “Playing” | |  |
| Playing individually | | The following activities that involve one player; running across pen, rolling, pivoting, tossing head, flopping, sliding, scampering, nudging |
| Playing socially | | The following activities that involve more players; e.g. running, pivoting, scampering, sliding across pen together, play fighting. |
| Playing with chew object | | (Energetically) shaking head with chew object in mouth, flipping chew object over, rolling ball over floor |
| “Manipulating pen mates” | |  |
|  | Ear biting | Chewing, nibbling or sucking the ear of a pen mate |
|  | Tail biting | Chewing, nibbling or sucking the tail of a pen mate |
|  | Belly nosing^2^ | Rubbing belly of a pen mate with ≥3 up and down movements of the snout or sucking the navel or skin of the abdominal area of a pen mate |
|  | Manipulating pen mates | Chewing, nibbling or sucking part of the body of a pen mate excluding ear, tail and abdominal area |
| Manipulating sow^1^ | | Chewing, nibbling or sucking part of the body of the sow including ear, tail, abdominal area and pulling hairs |

^1^Pre-weaning only.

^2^Post-weaning only.

**Supplementary Table S4.** Overview of response parameters and the associated statistical models

| **Variable** | **Experimental unit** | **Trans-formation** | **Test** | **Fixed effects** | **Random effects^2^** | **Covariate** |
| --- | --- | --- | --- | --- | --- | --- |
| Before weaning | | | | | | |
| Creep feed intake | Litter | Log | General linear mixed model  using spatial power covariance structure | Batch, period | Litter |  |
| Eater (yes/no) | Piglet |  | Generalized linear mixed model,  binary distribution, logit link function | Batch, day | Litter, litter within day |  |
| Average daily gain, BW | Piglet |  | General linear mixed model | Batch,  creep feed provision | Litter | Litter size |
| Uniformity in BW one day before weaning | Litter |  | General linear model | Batch,  creep feed provision |  |  |
| Behavior | Piglet |  | Generalized linear mixed model,  binomial distribution, logit link function, additional multiplicative overdispersion parameter | Batch,  creep feed provision, day, creep feed provision*day | Litter, litter within day |  |
| After weaning | | | | | | |
| Average daily gain, BW | Piglet |  | General linear mixed model | Batch, creep feed provision,  creep feed supplementation, provision*supplementation | Weaner pen, litter |  |
| Uniformity in BW at d14 post-weaning | Weaner pen |  | General linear model | Batch, creep feed provision,  creep feed supplementation, provision*supplementation |  | Uniformity in BW one day before weaning |
| Average daily feed intake, feed conversion ratio, fecal consistency score | Weaner pen |  | General linear model | Batch, creep feed provision,  creep feed supplementation, provision*supplementation |  |  |
| Average daily feed intake between d0-2^1^ | Weaner pen |  | General linear model | Creep feed provision,  creep feed supplementation, provision*supplementation |  |  |
| Average daily intake of creep feed supplement | Weaner pen |  | General linear model | Batch, creep feed provision |  |  |
| Duration of diarrhoea | Weaner pen |  | Generalized linear model  Poisson distribution, log link function, additional multiplicative overdispersion parameter | Batch, creep feed provision, creep feed supplementation, provision*supplementation |  |  |
| Behavior | Piglet |  | Generalized linear mixed model,  binomial distribution, logit link function, additional multiplicative overdispersion parameter | Batch, creep feed provision,  creep feed supplementation, provision*supplementation | Weaner pen, litter |  |
| Before and after weaning | | | | | | |
| Haptoglobin | Piglet | Log | General linear mixed model  using spatial power covariance structure | Batch, creep feed provision, day, provision*day | Piglet |  |

^1^For ADFI data between d0-2 post-weaning no batch effect was included as it was recorded in batch 2 only as result of technical difficulties in batch 1

^2^Litter was nested within batch and creep feed provision; weaner pen was nested within batch, creep feed provision and creep feed supplementation.

**Supplementary Figure S2.** Creep feed intake (back-transformed LS-means and their 95% CIs) and percentage of eaters (means ± SEM based on pen averages) before weaning in litters that were provided with creep feed from 2 days of age until weaning at 30 days of age. Eater = piglet scored eating creep feed from the feed trough or floor at least once per observation day of behavior (148, 144, 143 and 143 piglets from 12 litters at 11, 16, 22 and 27 days of age respectively). Superscripts without a common letter differ over time at *P* < 0.05. *P*-values < 0.05 are presented in bold.


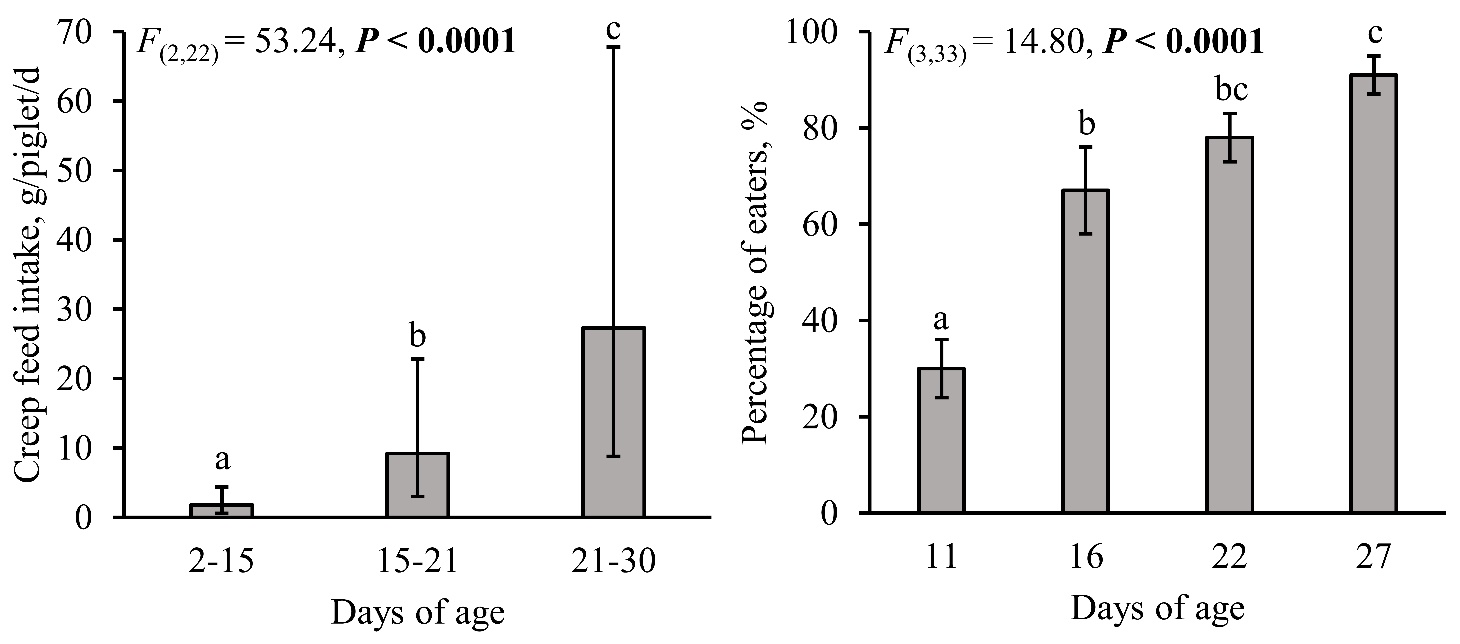

Supplement: Supplementary file 2 [file Data_Sheet_2.DOCX]
